# Contact Load on the Current-Carrying Tribological Performance of Copper–Graphite Composites

**DOI:** 10.3390/ma18102391

**Published:** 2025-05-20

**Authors:** Jiayu Ye, Nenghui Wang, Haihong Wu, Chuanfeng Wang, Xiao Kang

**Affiliations:** 1 CSSC Jiangxi Jiujiang Marine Equipment Co., Ltd., Jiujiang 332008, China; 2 School of Materials Science and Engineering, Central South University, Changsha 410083, China; 3 State Key Laboratory of Powder Metallurgy, Central South University, Changsha 410083, China

**Keywords:** wear, load, current-carrying, copper matrix composites

## Abstract

This study investigates the current-carrying tribological properties and wear mechanisms of copper–graphite composites under varying contact loads. Two copper–graphite composites with different graphite content were prepared using the pressure sintering method. Current-carrying tribological tests were conducted at three distinct contact loads. Scanning electron microscopy, X-ray diffraction, laser confocal microscopy, and pin-on-disk tribological testing were utilized to examine the current-carrying tribological properties and the worn morphologies of the materials. The results indicate that, under the three contact loads, the friction coefficient of the copper–graphite materials ranged from 0.3 to 0.5, the wear rate was on the order of 10^−13^ m^3^/(N·m), the average voltage drop varied between 0.7 and 1.6 V, and the average electrical noise ranged from 0.2 to 0.9 mV. The wear mechanism included delamination wear and a minor amount of abrasive wear, and the lubricating film formed on the surface was mainly composed of C, PbO, and CuO. Notably, copper–graphite composites with lower graphite content exhibited superior hardness, electrical conductivity, and relative density compared to those with higher graphite content. At a contact load of 0.31 N, the copper–graphite composite containing 30wt% graphite demonstrated the most favorable current-carrying tribological performance, characterized by the lowest wear rate (1.09 × 10^−13^ m^3^/(N·m)), voltage drop (0.943 V), and electrical noise (0.234 mV).

## 1. Introduction

As the essential component of rotating electrical connections, conductive slip rings demonstrate exceptional power and signal transmission capabilities, playing critical roles in aerospace, wind power generation, shipping, and various other sectors [1,2,3,4,5,6,7,8]. With the growing demands for power and signal transmission, coupled with the diversity of application scenarios, the development of conductive slip rings is increasingly oriented towards high power, enhanced stability, and extended lifespan [9,10,11].

Currently, researchers are concentrating on enhancing the performance of conductive slip rings through material selection and structural optimization [12,13,14,15,16]. Copper-graphite composites, recognized for their superior strength, self-lubricating properties, electrical conductivity, and thermal conductivity, are commonly employed in electrical contact materials [17,18,19]. Furthermore, researchers have determined that the preparation process, component ratio, and incorporation of modifying elements can significantly influence the properties of copper–graphite composites [20,21,22,23,24,25]. Among these factors, the graphite content directly affects the current-carrying friction and wear properties of copper–graphite composites [26,27,28,29,30,31,32,33]. Liu et al. [27] prepared four copper–graphite composites with various compositions and discovered that increasing the graphite content enhances wear resistance while diminishing electrical contact performance. The optimal overall performance was attained with a graphite content of 70%. Grandin et al. [28] discovered that increasing the copper content effectively reduces the contact resistance of copper–graphite composites. Following wear, a friction film primarily composed of graphite and Cu_2_O forms on the material surface. Furthermore, a higher Cu_2_O content in the friction film correlates with lower contact resistance. Zhu et al. [31] examined the wear mechanisms of copper–graphite composites and found that with a graphite content of 30%, the principal wear mechanism is adhesive wear. In contrast, at a graphite content of 25%, the wear mechanism shifts to a combination of adhesive wear and arc erosion. Additionally, contact load significantly affects the friction and wear properties of copper-based composites [34,35,36,37,38,39,40]. Wu et al. [34] investigated the dynamic behavior of arcs and surface damage mechanisms during current-carrying wear of copper-based composites under varying loads. They observed that as the load increases, the average friction coefficient decreases, the occurrence of arcs becomes more likely, and both the rate and energy of the arcs increase. This phenomenon leads to a transition from mechanical damage to electrical damage at the current-carrying friction interface. Wang et al. [35] studied the effect of load on the current-carrying friction performance of C2700, finding that as the load increases, both the friction coefficient and contact voltage drop decrease; however, excessively high loads result in increased wear. Zhang et al. [37] investigated the wear mechanisms of copper-based composites under different loads and found that at a load of 2 N, the material experiences only slight wear, characterized by fatigue and adhesive wear. Conversely, at a load of 50 N, microcracks and cavitation appear on the material surface, indicating that adhesive wear is the primary wear mechanism.

In this study, two copper–graphite composites with varying graphite contents were prepared, and current-carrying friction and wear experiments were conducted under different contact loads. By analyzing the wear characteristics of the brushes and their mating surfaces, the wear patterns of the brushes were investigated to identify copper–graphite brushes exhibiting low wear and exceptional electrical contact performance, along with their optimal contact loads. This research offers valuable insights for the preparation and selection of high-performance conductive slip rings.

## 2. Experimental

### 2.1. Composites

Due to the severe wear and high current demands of conductive slip rings in SPM applications, two copper–graphite brush variants were formulated, incorporating graphite mass fractions of 45% and 30%, respectively. Additionally, to enhance the hardness and tribological stability of these materials, a 3% mass fraction of lead was added to the copper–graphite mixture [41,42]. Table 1 outlines the precise compositions of these composite materials. For clarity, the two copper–graphite composites are designated as composites A and B.

The experimental materials included electrolytic copper powder (particle size: 10–50 μm, provided by Beijing Beike 2D materials Co., Ltd., Beijing, China), graphite powder (particle size: 50–100 μm, provided by Tianjin Gaoke New Materials Technology Co., Ltd., Tianjin, China), and lead powder (particle size: 10–50 μm, provided by Hebei TeBo Metal Materials Co., Ltd., Xingtai, China). All raw material powders had a mass fraction (purity) exceeding 99%.

Initially, the powders were precisely weighed, as detailed in Table 1, and uniformly mixed in a ball mill (Miqi, YXQM-4L, Changsha, China) at a ball-to-material ratio of 10:1 and a rotational speed of 200 r/min for 24 h, using 2 mm diameter copper balls. The resultant mixture was then pressed into a rectangular block measuring 4 mm × 4 mm × 25 mm at a pressure of 300 MPa for 15 s, using a precision servo hydraulic press (Riyi, FBSY-C05, Taizhou, China). Subsequently, the pressed block was sintered in a hydrogen furnace (Kejing, KSL-1200X, Hefei, China), held at 900 °C for 1 h, and then cooled within the furnace.

### 2.2. Counterpart Materials

In light of the requirement for superior electrical and thermal conductivity in counterpart materials [43,44], a QCr0.8 copper–zirconium alloy with a hardness of 150 HV was selected and machined into disks with a diameter of 70 mm and a thickness of 5 mm.

## 3. Testing

**Figure 1:**
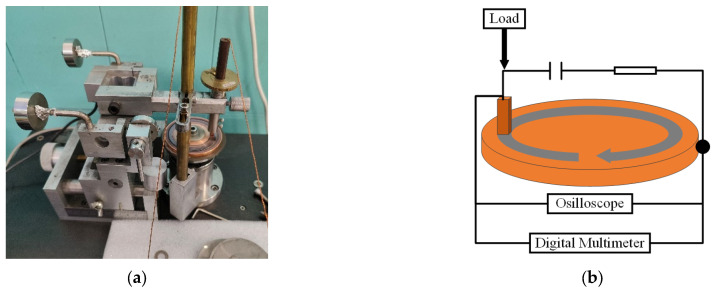
Schematic diagram of current-carrying tribological performance testing equipment. (**a**) Digital picture and (**b**) schematic diagram.

A self-made pin-on-disk tribological tester was used in this experiment to test the current-carrying tribological properties, as illustrated in Figure 1. The test was conducted at a rotational speed of 100 r/min, a friction distance of 5000 m, a current of 6 A, and a load of 0.3 N. During the friction process, the device could automatically record the real-time friction coefficient, while the voltage drop and electrical noise curves were collected and analyzed using an oscilloscope (Yokogawa, DL 850, Tokyo, Japan). The wear rate of the material was calculated using Equation (1):

(1) $$\delta =\frac{m_{1}-m_{2}}{\rho \cdot F\cdot L}$$

where *δ* is the wear rate, *m*_1_ and *m*_2_ are the masses of the material before and after wear, measured using an electronic analytical balance (Sartorius, TE214S, Göttingen, Germany) with a precision of 0.0001 g, *ρ* is the material density, *F* is the applied normal load, and *L* is the sliding distance.

Density testing was conducted using the water immersion method based on Archimedes’ principle. The density was determined by measuring the changes in water volume and the mass of the material before and after submersion in water. Hardness testing was performed using a nanoindentation tester (Nanotest Vantage, Wrexham, UK). Electrical conductivity was calculated from the resistivity measured via the bridge method. Scanning electron microscopy (Tescan, Mira4, Brno, Czech Republic) combined with an energy dispersive spectrometer (Oxford, UK), X-ray diffraction (Rigaku SmartLab SE, Tokyo, Japan), and a laser confocal microscope (Keyence, VK-X200, Osaka, Japan) were used to analyze the worn surfaces of the copper–graphite materials and the counterpart disks.

## 4. Results

### 4.1. Microstructure

**Figure 2:**
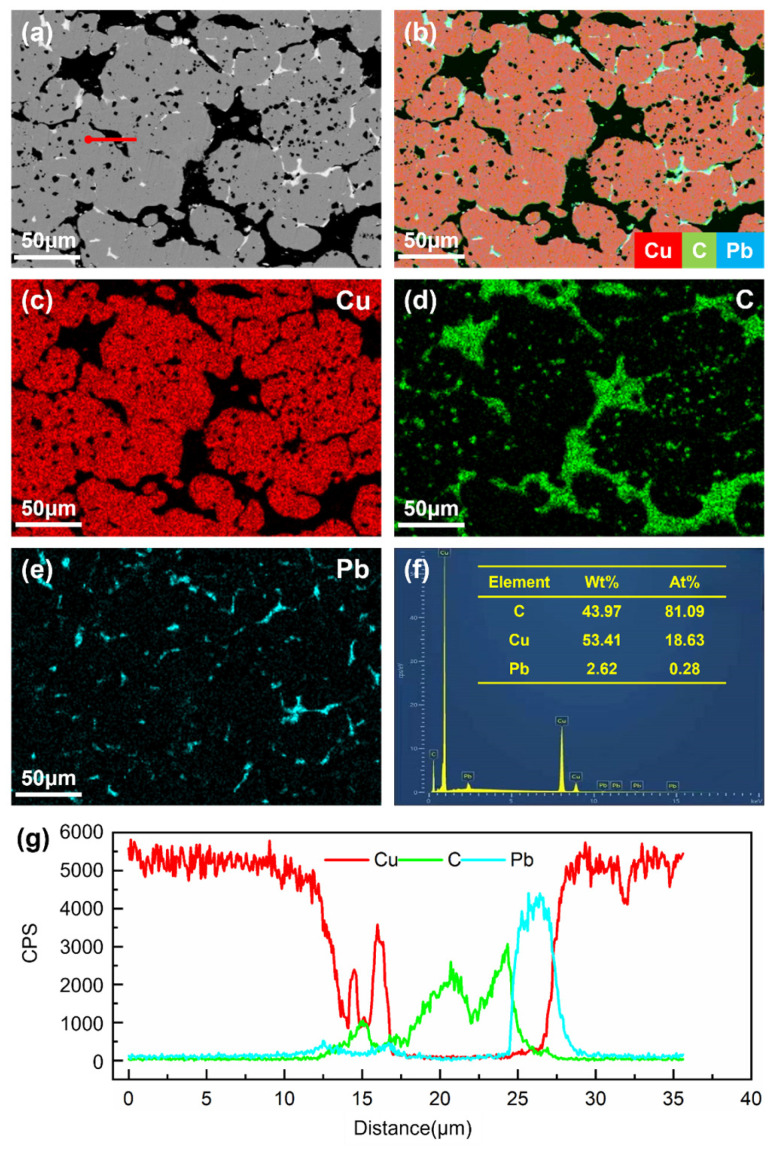
BSE image (**a**), element distribution maps (**b**–**e**), and EDS analysis results (**f**,**g**) of composite A.

**Figure 3:**
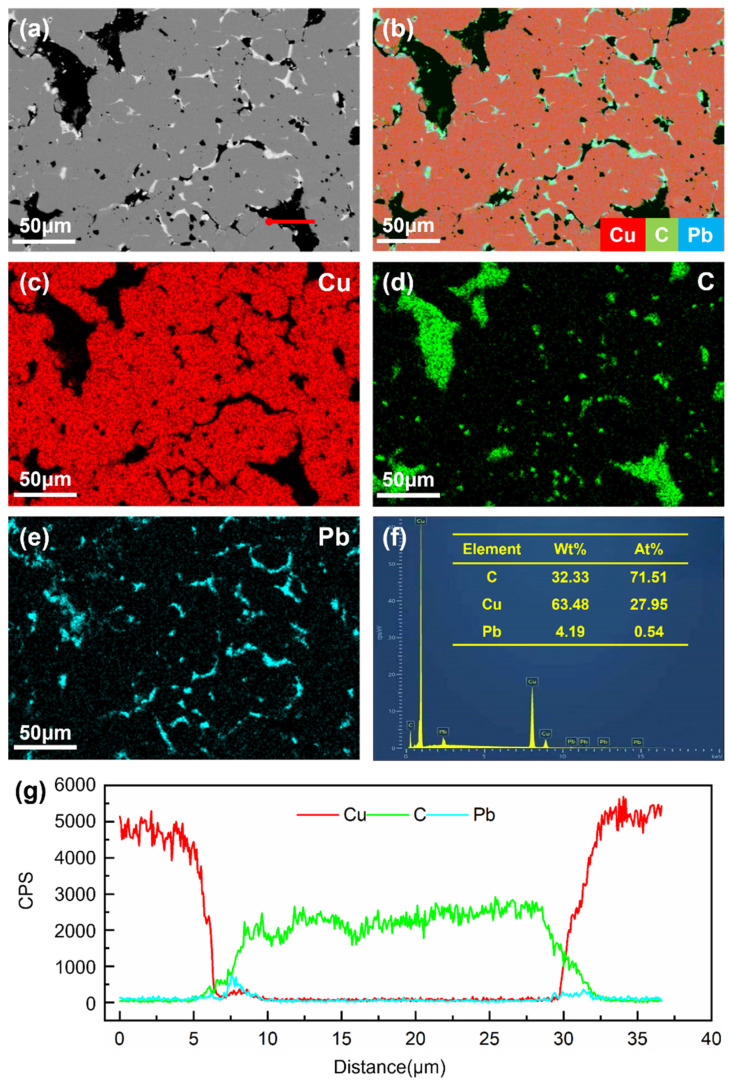
BSE image (**a**), element distribution maps (**b**–**e**), and EDS analysis results (**f**,**g**) of composite B.

Figure 2 and Figure 3 display the backscattered electron (BSE) images, elemental distribution maps, and EDS analysis results of composites A and B, respectively. In Figure 2b and Figure 3b, the red phase corresponds to the copper matrix, the green phase represents graphite particles, and the blue phase denotes lead particles. It can be observed that both graphite and lead particles exhibit an inhomogeneous, vermicular (worm-like) distribution within the copper matrix. The line scan profiles along the red lines in Figure 2g and Figure 3g reveal overlapping regions in the Cu, C, and Pb intensity curves, indicating diffusion phenomena during the composite fabrication process. Additionally, EDS analysis demonstrates that the mass fractions of graphite in composites C1 and C2 are approximately 44% and 32%, respectively. These results confirm that the actual compositions of the synthesized composites align with the design specifications, ensuring the reliability of subsequent experimental data.

### 4.2. Basic Properties

**Figure 4:**
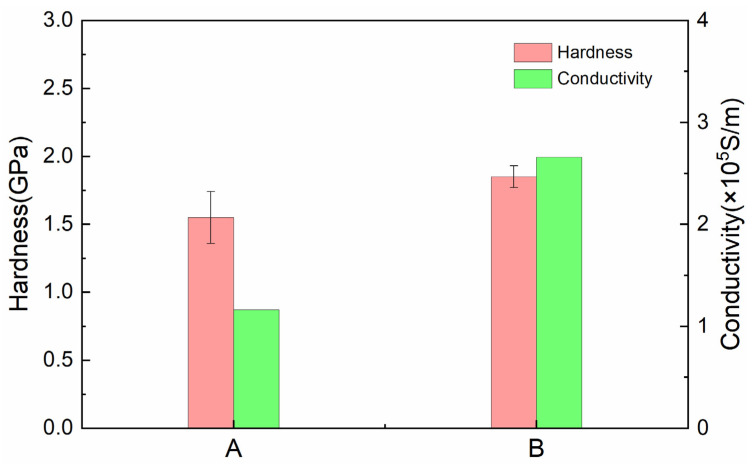
Hardness and conductivity of composites A and B.

Figure 4 shows the hardness and electrical conductivity of composites A and B, and Table 2 presents the measured density, theoretical density, and relative density of composites A and B. The results reveal that composite B has higher hardness (1.85 GPa), electrical conductivity (2.66 × 10^5^ S/m), and relative density (82.07%) compared to composite A’s hardness (1.55 GPa), electrical conductivity (1.16 × 10^5^ S/m), and relative density (68.75%). These findings indicate that increasing graphite content reduces the hardness, electrical conductivity, and relative density of copper–graphite composites. This phenomenon may occur because graphite, as a soft phase (Mohs hardness 1–2), weakens the continuity and load-bearing capacity of the copper matrix, and its lamellar structure leads to weakened interfacial bonding and introduces pores, thereby increasing the porosity of the composites. Additionally, the electrical conductivity of graphite (approximately 1 × 10^4^ S/m) is significantly lower than that of copper (5.8 × 10^7^ S/m), and its non-uniform distribution disrupts the conductive network of copper and intensifies electron scattering. Pores, acting as insulating regions, further hinder current conduction. Ultimately, higher graphite content results in lower hardness, electrical conductivity, and relative density in the composites [45,46].

### 4.3. Current-Carrying Tribological Properties

**Figure 5:**
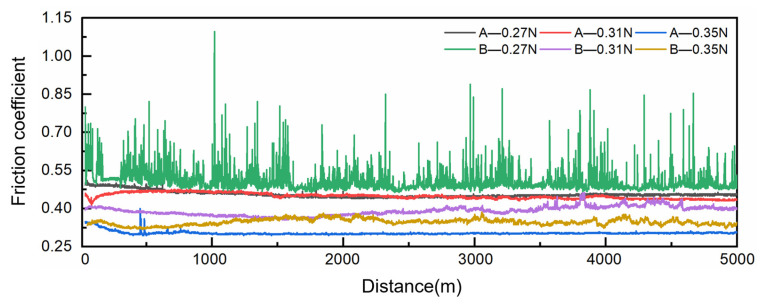
Friction coefficient curves of composites A and B under different loads.

**Figure 6:**
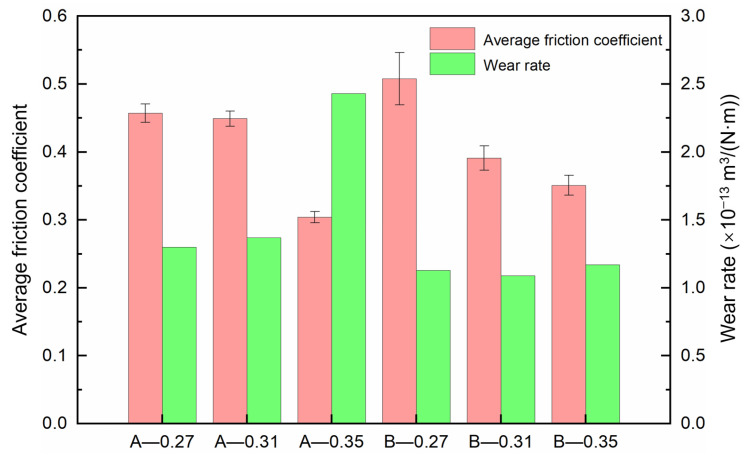
The average friction coefficient and wear rate of composites A and B under different loads.

Figure 5 shows the friction coefficient curves of composites A and B under different loads, and Figure 6 presents their average friction coefficients and wear rates. It can be observed that, except for the large fluctuations in the friction coefficient curve of B under a load of 0.27 N, the other friction coefficient curves are relatively stable, fluctuating within the range of 0.3 to 0.5. This phenomenon may be attributed to insufficient load, which hinders the adequate release of the lubricating graphite phase in material B, thereby preventing the formation of a continuous lubricating film on its surface. In contrast, material A exhibits little fluctuation in the friction coefficient curve under a load of 0.27 N due to its higher graphite content compared to B. The abundant lubricating phase in A facilitates the formation of a continuous lubricating film. These observations align with the experimental findings of Ma et al. [47], who reported that inadequate activation of copper–graphite composites under low loads leads to significant fluctuations in friction coefficient curves. At a load of 0.35 N, the average friction coefficients of A and B are the lowest, at 0.304 and 0.351, respectively. The wear rates are all in the order of 10^−13^ m^3^/(N·m). The wear rate of A gradually increases with increasing load, with the lowest wear rate of 1.30 × 10^−13^ m^3^/(N·m) under a load of 0.27 N. For B, the wear rates exhibit no significant differences, and the lowest wear rate of 1.09 × 10^−13^ m^3^/(N·m) is observed under a load of 0.31 N.

**Figure 7:**
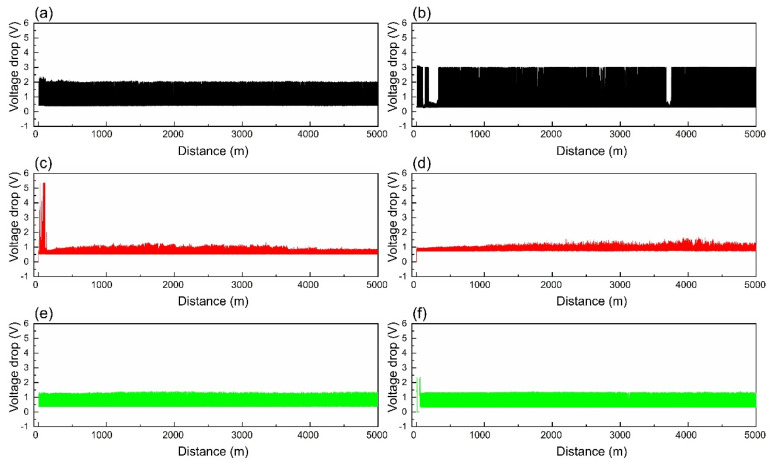
Voltage drop curves of composites A and B under different loads: (**a**) A—0.27 N; (**b**) B—0.27 N; (**c**) A—0.31 N; (**d**) B—0.31 N; (**e**) A—0.35 N; (**f**) B—0.35 N.

**Figure 8:**
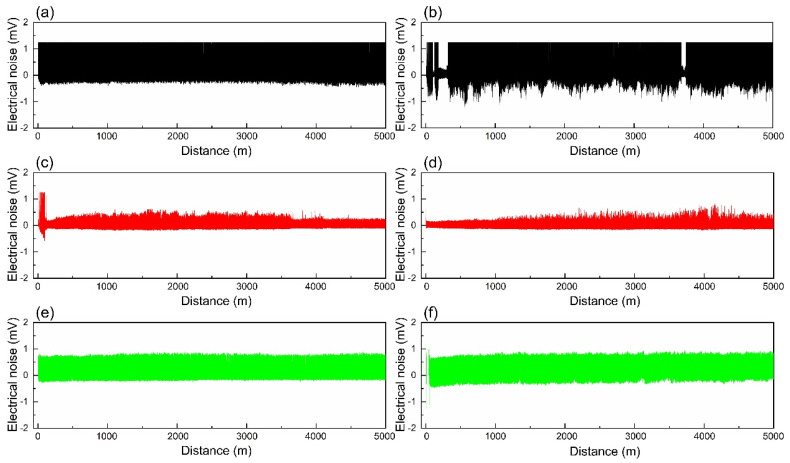
Electrical noise curves of composites A and B under different loads: (**a**) A—0.27 N; (**b**) B—0.27 N; (**c**) A—0.31 N; (**d**) B—0.31 N; (**e**) A—0.35 N; (**f**) B—0.35 N.

**Figure 9:**
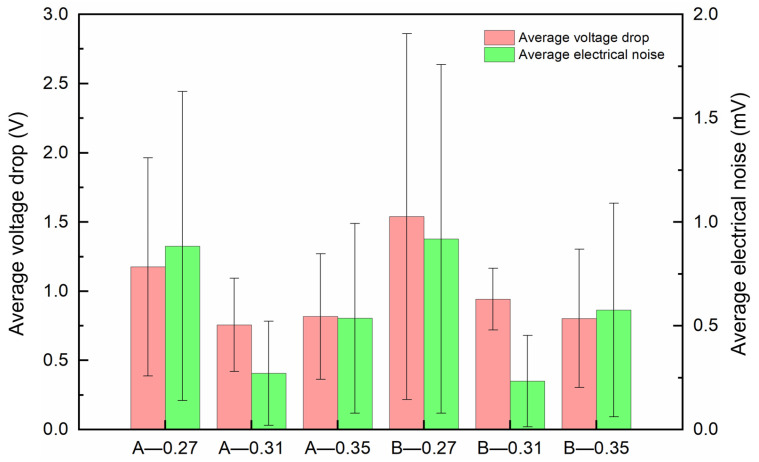
The average voltage drop and electrical noise of composites A and B under different loads.

Figure 7 and Figure 8 depict the voltage drop curves and electrical noise curves of composites A and B under different loads, respectively, and Figure 9 shows their average voltage drops and average electrical noise. It can be seen that when the contact load is 0.27 N, the voltage drop and electrical noise curves of both A and B exhibit large fluctuations. The average voltage drops of A and B are 1.180 V and 1.360 V, respectively, and the RMS values of electrical noise are 0.884 mV and 0.918 mV, respectively. When the contact load is 0.31 N, the voltage drop and electrical noise curves of A show significant fluctuations initially but stabilize thereafter, while the curves of B remain stable throughout. The average voltage drops of A and B are 0.758 V and 0.943 V, respectively, and the average electrical noise values are 0.271 mV and 0.234 mV, respectively. At a contact load of 0.35 N, the fluctuations in the voltage drop and electrical noise curves of both A and B are slightly smaller than those at 0.27 N, with average voltage drops of 0.817 V and 0.803 V, and average electrical noise of 0.536 mV and 0.576 mV, respectively. This indicates that both A and B exhibit the best electrical contact stability at a load of 0.31 N.

### 4.4. Wear Characteristics

**Figure 10:**
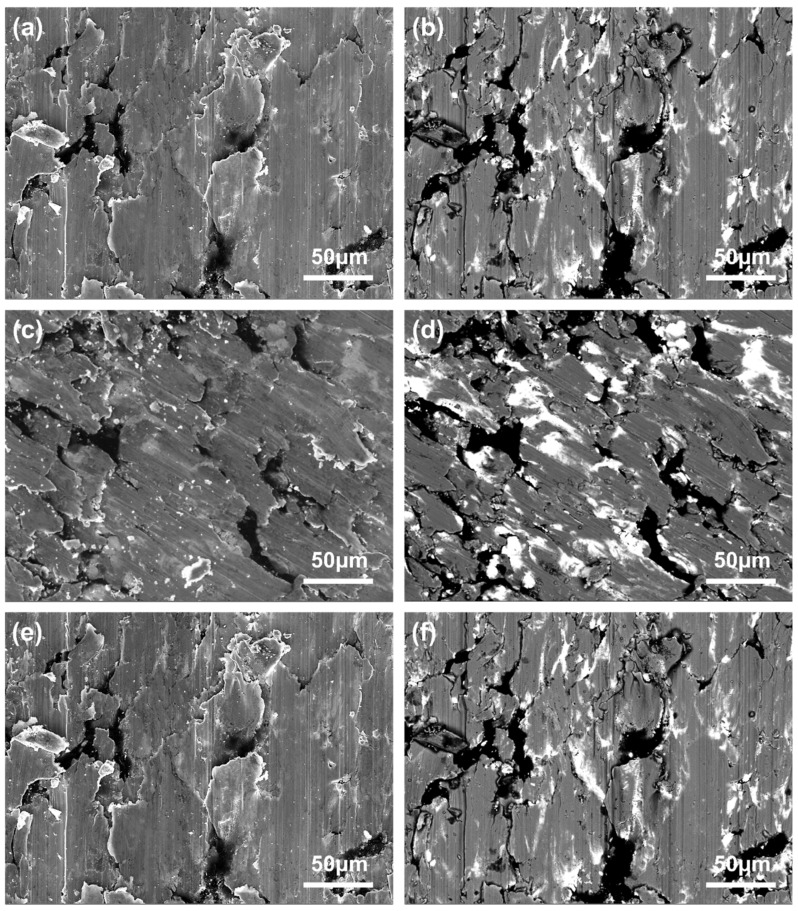
SEM images of 0.27 N (**a**,**b**), 0.31 N (**c**,**d**), and 0.35 N (**e**,**f**) of worn surface of composite A.

**Figure 11:**
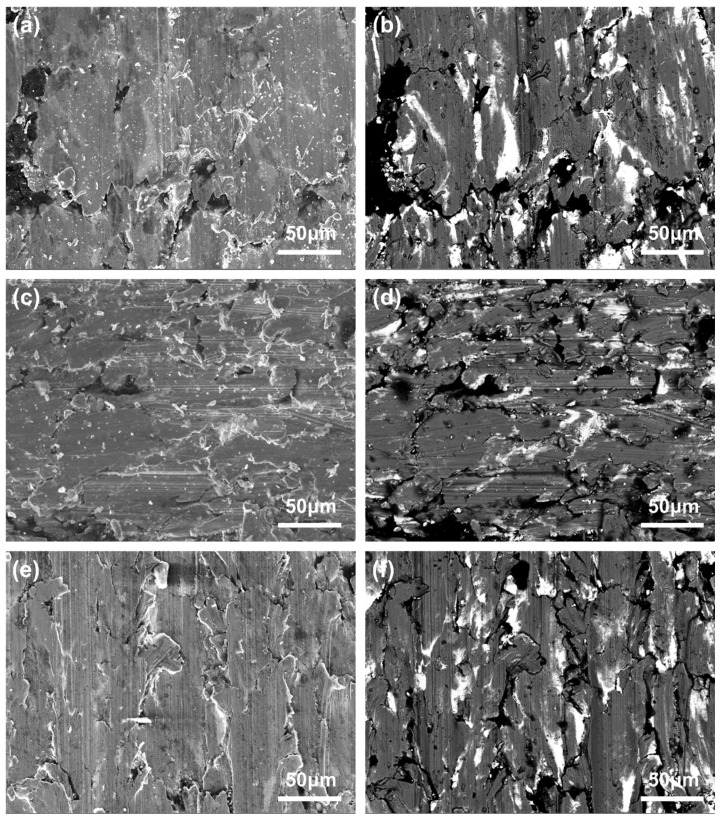
SEM images of 0.27 N (**a**,**b**), 0.31 N (**c**,**d**), and 0.35 N (**e**,**f**) of worn surface of composite B.

Figure 10 and Figure 11 present complementary scanning electron microscopy (SEM) analyses of the worn surfaces of composites A (Figure 10) and B (Figure 11), comparing secondary electron (SE, left) and backscattered electron (BSE, right) imaging modes. The SE images emphasize fine-scale surface topography through electron emission sensitivity to surface curvature, while the BSE images reveal sub-surface compositional variations based on atomic number contrast, with brighter regions indicating higher average atomic number phases. This dual-mode characterization is critical for elucidating wear mechanisms in these composites. Under varying loads, the worn surfaces of both A and B exhibit a limited number of grooves and wear debris, alongside numerous layered material structures and minor cracking. This observation suggests that the primary wear mechanism of the brushes involves delamination wear with some abrasive wear. As the load increases from 0.31 N to 0.35 N, the worn surfaces of the brushes become smoother and more uniform, which aligns with the decreasing trend of the friction coefficient. Additionally, according to previous research findings [48], the brush surfaces are coated with a lubricating film composed of a mixture of graphite and lead, providing effective lubrication.

**Figure 12:**
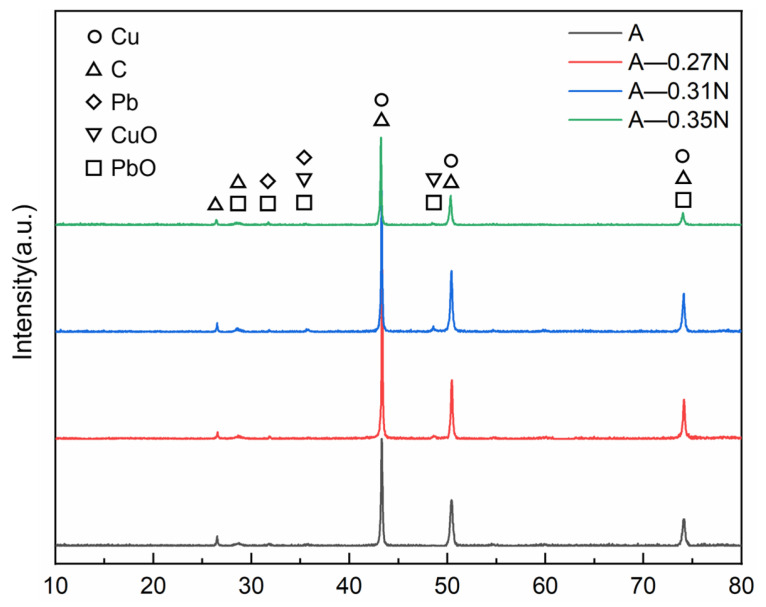
XRD pattern of worn surface of composite A.

**Figure 13:**
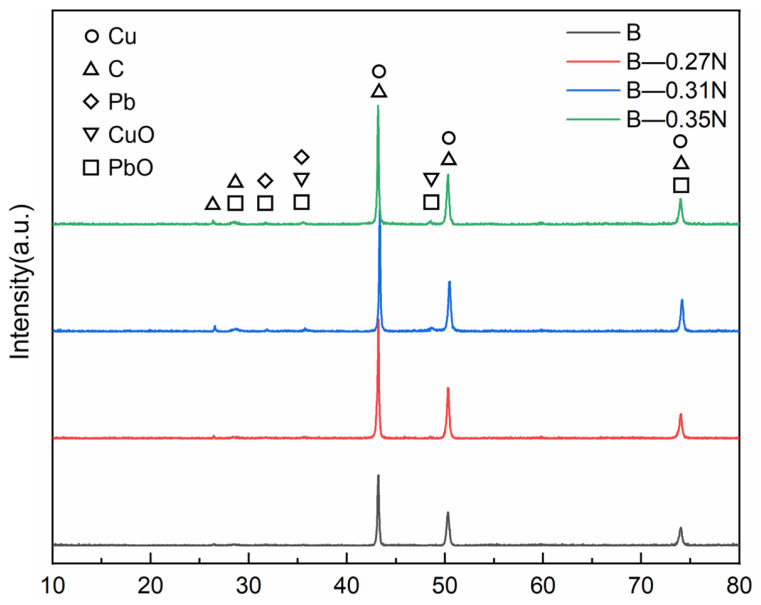
XRD pattern of worn surface of composite B.

Figure 12 and Figure 13 show the XRD patterns of the composite material surface before and after wear. It can be observed that the diffraction peak positions of Cu, C, and Pb remain unchanged, indicating that current-carrying wear does not alter the crystal orientation. The figures confirm that a lubricating film containing PbO and CuO forms on the composite surface during wear. This is attributed to frictional heat triggering oxidation reactions on the material surface, generating PbO and CuO. PbO, with a layered crystal structure similar to graphite and low shear strength, forms a shear-slip layer at the friction interface, reducing the friction coefficient. Meanwhile, CuO, which has higher hardness (approximately 3 GPa), enhances the surface resistance to plastic deformation and mitigates abrasive wear in small amounts [41,42,49].

For composite B under a load of 0.27 N, the diffraction peak intensities of solid lubricating phases (C, PbO, and CuO) in the surface lubricating film are lower than those in other groups. This further demonstrates insufficient release of lubricating phases in the composite under this condition, consistent with the friction coefficient curve results shown in Figure 5.

**Figure 14:**
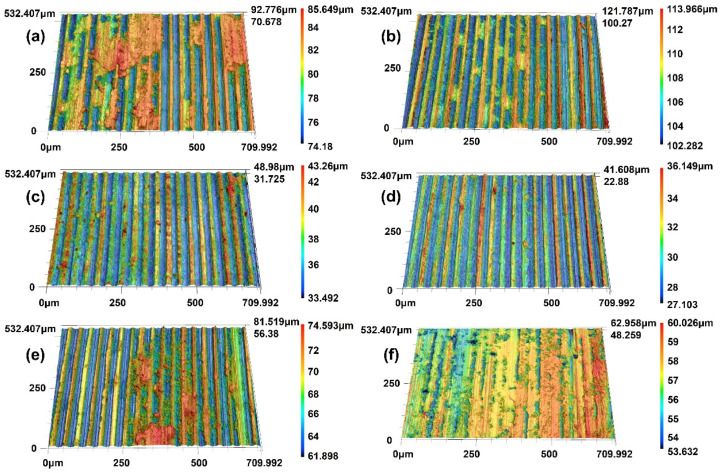
Three-dimensional contour of the worn surface of the counterpart disks: (**a**) A—0.27 N; (**b**) B—0.27 N; (**c**) A—0.31 N; (**d**) B—0.31 N; (**e**) A—0.35 N; (**f**) B—0.35 N.

Figure 14 presents the three-dimensional profile of the counterpart disc surface after brush wear, acquired via laser confocal microscopy to enable enhanced analysis of wear mechanisms. At a contact load of 0.27 N, the counterpart disc worn by the brush exhibits numerous plowing grooves covered with a significant number of debris, indicating that the primary wear mechanisms are abrasive and adhesive wear. As the contact load increases to 0.31 N, the depth of the plowing grooves on the counterpart disc surface becomes shallower, and the debris coverage diminishes, suggesting that the predominant wear mechanism shifts primarily to abrasive wear. When the contact load reaches 0.37 N, the counterpart disc surface still displays plowing grooves with substantial debris coverage, indicating that the main wear mechanisms are abrasive and adhesive wear. In contrast, the counterpart disc worn against brush B presents a larger area covered with debris and a smoother surface.

## 5. Conclusions

1. Composite A, with a higher graphite content, demonstrates enhanced lubrication performance, enabling the formation of stable lubrication films under lower contact loads. However, the increased graphite content also results in reductions in material hardness, electrical conductivity, and relative density. In contrast, composite B exhibits superior performance in terms of hardness and electrical conductivity, rendering it more suitable for electric brush applications.
2. Under a contact load of 0.31 N, both composite materials exhibited optimal electrical contact stability, with minimized voltage drop and electrical noise fluctuations. While the friction coefficient tended to decrease with increasing load, excessively high loads led to exacerbated wear.
3. At a contact load of 0.31 N, the copper–graphite brushes exhibited the best current-carrying friction and wear performance, with the lowest wear rate, voltage drop, and electrical noise. Specifically, for brush B, the average friction coefficient, wear rate, average voltage drop, and average electrical noise were 0.406, 1.09 × 10^−13^ m^3^/(N·m), 0.943 V, and 0.234 mV, respectively.
4. The wear mechanisms of the two copper–graphite brushes under different loads were primarily delamination wear with a small amount of abrasive wear. As the contact load increased, the brush wear surfaces gradually became smoother and more even, and the lubricating film formed on the surface was mainly composed of C, PbO, and CuO.

## Figures and Tables

**Table 1:** Chemical composition of copper–graphite composites (mass fraction, %).

Number	C	Pb	Cu
A	45	3	52
B	30	3	67

**Table 2:** Density of composites A and B.

Number	Measured Density (g/cm^3^)	Theoretical Density (g/cm^3^)	Relative Density (%)
A	5.6	3.85	68.75
B	5.8	4.76	82.07

## Data Availability

The original contributions presented in this study are included in the article. Further inquiries can be directed to the corresponding author(s).
